# Local Anæsthesia

**Published:** 1890-02

**Authors:** 


LOCAL ANÆSTHESIA WITH SELTZER WATER.—Dr. Voituriez (*Journ. de sic. med. de Lille*) recommends the use of siphons of seltzer water for this purpose, the jet being held at about four inches from the region to be anæsthetized. He uses at first two or three bottles of seltzer, which causes an anæsthesia that will last for four or five minutes, after which a small additional quantity will suffice to prolong the effect.—*American Journal of Pharmacy*, Dec., 1889.

